# Preparation for Cancer Treatment: A Cross-Sectional Study Examining Patient Self-Reported Experiences and Correlates

**DOI:** 10.3390/ijerph191610167

**Published:** 2022-08-17

**Authors:** Heidi Turon, Breanne Hobden, Kristy Fakes, Matthew Clapham, Anthony Proietto, Rob Sanson-Fisher

**Affiliations:** 1 Health Behaviour Research Collaborative, School of Medicine and Public Health, Faculty of Health and Medicine, University of Newcastle, Callaghan, NSW 2308, Australia; 2 Priority Research Centre for Health Behaviour, Faculty of Health and Medicine, University of Newcastle, Callaghan, NSW 2308, Australia; 3 Hunter Medical Research Institute, New Lambton, NSW 2305, Australia; 4 Clinical Research Design and Statistics, Hunter Medical Research Institute, New Lambton, NSW 2305, Australia

**Keywords:** cancer, treatment preparation, cross-sectional

## Abstract

Given the significant physical and psychosocial side-effects cancer treatment has on individuals, it is important to ensure patients receive adequate preparation prior to treatment. The purpose of this study was to explore, among Australian oncology patients, (i) the self-reported treatment preparation information they received; and (ii) the patient characteristics associated with the treatment preparation information received. Patients in the early stages of cancer treatment were invited to complete a survey exploring their receipt of information about treatment preparation. Items assessed patients’ self-report of whether they had received information about the treatment process. A total of 165 participants completed the survey. Patients most frequently reported receiving information about how they might feel physically (94%) and what side effects to watch for (93%). One in five patients reported not receiving information about how to cope with any stress or worry related to treatment. Females reported receiving significantly fewer items of care compared to males (*p* = 0.0083). This study suggests that while self-reported preparation for cancer treatment is generally high, components of preparation related to psychosocial concerns could be improved. Survey data could be used as a feedback tool for centres to monitor delivery of care.

## 1. Introduction

Cancer treatment, regardless of tumour type, is often complex, time-intensive and associated with significant side effects, causing a high degree of physical and emotional burden for patients [1,2]. Patient education has been identified as a key component of patient-centred care by the Institute of Medicine [3]. To meet the requirements of informed consent for treatment, it is essential that patients be adequately prepared for their planned medical intervention.

Patients with cancer have reported information about treatment as a high priority [4]. Patients who are well informed about cancer treatment have reported lower anxiety throughout treatment [5,6,7], as well as being more likely to adhere to treatment plans and medication [8] and achieve improved health outcomes [9,10]. For example, interventions to improve patient preparation have demonstrated positive outcomes within cancer care. A systematic review examining patient preparation for chemotherapy and radiotherapy interventions found these were associated with improvements in patient knowledge, physical symptoms, cost and psychological outcomes or quality of life [11]. Another systematic review examined education interventions for pre-operative patients with cancer and demonstrated face-to-face interventions resulted in benefits to anxiety levels, patient satisfaction, knowledge and healthcare costs [12]. Despite the demonstrable benefits of patient education for cancer treatment, it is unclear whether these evidence-based techniques have been adopted as part of care provisions in routine practice.

Assessment of patients’ perceptions of the quality of care they receive in healthcare environments is often measured by patient experience surveys, with specific cancer care surveys available [13,14,15]. These surveys are designed to capture a broad range of care experiences and rarely capture details of patient experiences of preparation for cancer treatment. More nuanced approaches to measuring patient experiences of preparation are required. Further, the limited number of studies that have examined treatment preparation experience in patients with cancer suggests that there is scope for improvement in patient preparation, with patients commonly reporting being underprepared for treatment and its effects [16,17]. There is also a paucity of data available exploring whether certain patient groups are more likely to report being underprepared. As such, the need for further research into this area was identified via a patient-reported survey.

This article aims to examine, among oncology patients who have recently commenced cancer treatment, the (i) self-reported treatment preparation information they received; and (ii) the patient characteristics associated with the treatment preparation information received.

## 2. Materials and Methods

### 2.1. Design and Setting

A descriptive cross-sectional study was conducted in four oncology treatment centres in NSW, Australia. Three of the treatment centres were located in public hospitals (one in a rural location) and one was in a private hospital.

### 2.2. Patient Eligibility Criteria

Individuals attending a participating treatment centre, who met the following criteria, were eligible to participate in the study: aged 18 years or older; have a confirmed diagnosis of cancer; able to complete a survey in English; and able to provide informed consent. Patients who were perceived to be too ill by recruitment staff were not approached.

### 2.3. Measures

*Experiences of preparation for cancer treatment:* Participants completed the “Treatment Preparation” module of the System for Patient Assessment of Cancer Experiences (SPACE). Data describing the development of SPACE have been previously reported [18]. Briefly, SPACE was designed to capture patient experiences of care each time they attended the clinic. Patients completed several short, branching questions, which directed them to the most relevant module for their current stage in the treatment trajectory. Patients were able to complete the survey each time they attended the clinic but could only complete each module once. Patients who were receiving their first treatment or had commenced treatment in the last week, received a module exploring their treatment preparation. As part of this module, patients were asked 11 questions regarding their preparation for their current cancer treatment. An example item is:

“Before starting treatment, did you receive information about what would happen on the first day of treatment?”

The response scale for all items were: “Yes, and I wanted this”; “Yes, but I didn’t want this”; “No, but I wanted this”; or “No, but I didn’t want this”. For the purposes of analysis, responses were grouped as either “Yes” or “No” as we were primarily interested in whether participants received care consistent with previously identified best-practice care components.

*Sociodemographic and disease characteristics:* Patients self-reported their age, gender, highest level of education, country of birth, cancer type, cancer stage at diagnosis and how long ago they were diagnosed.

### 2.4. Procedure

A trained research assistant approached eligible patients in the outpatient clinic while waiting for their appointment or while receiving treatment as a day patient. The research assistant gave a verbal overview of the study and provided patients with a study information statement. Consenting patients then initiated the survey on a computer tablet. Implied consent was obtained through initiation and completion of the survey. The survey was administered using the software survey program QUON [19]. QUON is a custom-built web application designed for the delivery of online surveys. It allows for complex branching, a wide range of question and input types, and allows data to be stored locally, providing additional data security and privacy. The survey module took less than 3 min to complete and data was collected between October 2017 and March 2018. The study was approved by an institutional ethics committee (HREC/16/HNE/249), as well as the governance committees of each participating hospital.

### 2.5. Data Analysis

Frequencies and percentages were calculated for patient demographics and for each of the survey items. The items for which participant indicated “Yes” were then summed to give each participant a score from 0 to 11, which are hereafter referred to as ‘care scores’. Missing items were imputed using the mean if 5 or less were missing (i.e., <50% of total items available). A linear regression was conducted to assess the association between patient characteristics and the care scores. The parameter estimates with 95% confidence intervals and *p*-values from type III tests were calculated for each covariate to test the overall significance. As the scores were skewed to the higher end of the scale, residual analysis showed a decreasing model error variance as the predicted score increased. This is a possible violation of linear regression assumptions, so heteroscedasticity-consistent standard errors (Huber–White) were used and are reflected in the confidence intervals and *p*-values. All statistical analyses were completed in SAS v9.4 (SAS Institute, Cary, NC, USA).

## 3. Results

Patients were approached on 1481 occasions, in which 1379 involved an eligible patient (93%). A total of 985 patients consented to completing the SPACE survey on 1143 occasions (83% consent rate). Of the 985 consenters, 168 received the treatment preparation module of SPACE, and 165 had completed at least 6 out of 11 care items (98% completion rate) and were included in the results. The remaining consenters completed other modules of the SPACE survey, which are reported elsewhere [20]. The mean age of the participants was 62.6 years (SD ± 13.1) and 57% were female. Breast and haematological cancers were the most common diagnoses (25% and 19%, respectively). The sociodemographic and disease characteristics of the participants can be found in Table 1.

Overall, participants reported high rates of receiving treatment preparation information (Table 2). The types of information most frequently reported as being provided to patients included information related to the physical effects of treatment, including, ‘how they might feel physically’ (94%); ‘side effects to watch out for’ (93%); ‘who they can contact if unexpected side effects arise’ (93%); and ‘the best way of managing side effects’ (91%). The item least frequently endorsed was ‘how to cope with any stress or worry’, which was not received by 20% of participants. Over half (*n* = 110, 67%) of the participants reported receiving all 11 items of care. The mean number of items of care received was 9.83 (SD ± 2.34).

When examining the sociodemographic and disease characteristics associated with the care scores, gender was the only significant variable. On average, females reported receiving 1.34 less items of care (out of 11) than males (95% CI: −2.28 to −0.41, *p =* 0.0083). Results of the linear regression can be found in Table 3.

## 4. Discussion

This study explored patients’ experience of preparation for cancer treatment, in terms of their self-reported receipt of a number of care items identified as important to the delivery of high-quality care. These findings are similar to other recent research [21], and suggest that, overall, most patients report a high level of quality of care in relation to preparation for cancer treatment. For example, the most recently reported results from the Outpatient Cancer Clinics Survey [22], for patients who had attended a public outpatient cancer clinic in New South Wales in 2020, indicate that a high proportion of patients (87%) rated their care at cancer clinics as ‘very good’. This result suggests that the treatment centres involved in this study are providing patients with the majority of treatment information and preparation, based on best evidence.

Psychosocial aspects were the lowest endorsed as being received, with information about coping with stress and worry reported as not being received by 20% of participants. This is consistent with findings from a sample of Australian haematological cancer patients [23], and supports findings from unmet-needs studies, highlighting that psychosocial concerns are often rated most prominently as being unaddressed [24,25]. Psychosocial issues are important to address, as raised anxiety prior to medical procedures, including cancer treatment, has been suggested to be a predictor of pain, anxiety and use of medication during the actual procedure [26], and has also been reported to increase patients’ post-procedural pain and complications, delaying their recovery [27,28,29,30]. A number of effective strategies to prepare patients for treatment have been developed, which could be beneficial to those with psychosocial concerns. For example, increased provision of psychosocial preparation strategies before undergoing a medical procedure has been suggested as warranted for some subgroups of patients in relation to medical imaging procedures, particularly those with circulatory conditions and neoplasms [31]. Strategies such as discussion of the patient’s emotions, relaxation training (e.g., breathing exercises) or cognitive coping strategies [10,32] may also be of benefit to this population.

Compared to males, females reported receiving significantly less items of care. This finding aligns with previous research and quality assurance data that has demonstrated health inequities for females [33,34], including in cancer care [35]. Unconscious bias, stereotyping and gender biases are likely to play a role in this healthcare disparity [36]. For instance, research has indicated that providers are more likely to attribute heart problems in women reporting stress to psychological causes, while heart problems in men reporting stress were more likely to be perceived as physiological issues [37]. Disparities have also been reported in terms of administering medication and hospital admission for women who have experienced heart attacks [38]. In addition to unconscious bias in providers, women receiving treatment may not feel adequately empowered to be assertive in their healthcare preferences, which is interlaced with societal gender-related norms [39]. While the overall care quality in this study was relatively high, females reporting lower items of care indicates they are less likely to receive the benefits associated with optimal treatment preparation.

### 4.1. Limitations

It should be noted that our sample size was relatively small, and as such would not have had enough power to detect small effects. It is possible that a number of other characteristics may influence the care patients receive when being prepared for cancer treatment, such as self-efficacy, health literacy, cultural and linguistic background and the type of treatment patients are receiving. While we did not examine these factors in the current study, they would be a worthy focus of ongoing research. Finally, our assessment of the care provision was based on self-report, which can be subject to a range of bias. We minimised recall bias by ensuring only patients who were at the commencement of treatment completed this survey; however, future studies may wish to explore alternative research methods, such as recording consultations between the patient and clinician, to verify what information has been provided.

### 4.2. Future Research

While this research identified that the majority of cancer patients reported a high level of care related to preparation for treatment, our findings indicate that females report less items of care on average than males. Given gender differences in cancer care quality is a relatively limited area of research, this warrants further investigation to explore care perceptions in greater detail. We did not collect information about the gender of the treating clinician, and it would be interesting to explore if this has any impact on the perceived items of care received. Although beyond the scope of this study, future research could explore patients’ level of understanding of the information received. Finally, replication of this study in a larger sample of cancer treatment centres with greater representation of rarer cancer types would be valuable to determine the generalisability of these findings.

## 5. Conclusions

In summary, this study provided valuable insights to those involved in preparing patients for treatment, including the need for better psychosocial preparation. Our findings also suggest that staff should be particularly attentive to females to ensure the information presented is comprehensive and that they feel well informed. This module of SPACE has the potential to be used as an audit and feedback tool by cancer treatment centres to track patient perceptions of preparation.

## Figures and Tables

**Table 1:** Participant sociodemographic and disease characteristics (*n* = 165).

Variable		Total *n* (%)
Gender	Female	94 (57%)
	Male	71 (43%)
Highest level of education	Primary school	6 (3.8%)
	High school	79 (50%)
	Trade or vocational training	47 (30%)
	University degree	27 (17%)
Country of birth	Australia	126 (78%)
	United Kingdom	14 (8.7%)
	New Zealand	7 (4.3%)
	Other	14 (8.7%)
Type of cancer	Breast	41 (25%)
	Haematological/blood (e.g., lymphoma, leukaemia, myeloma)	30 (19%)
	Lung	20 (12%)
	Colorectal/bowel	13 (8.1%)
	Melanoma	11 (6.8%)
	Prostate	5 (3.1%)
	Brain	4 (2.5%)
	Other	37 (23%)
Stage of cancer at diagnosis	Early	69 (43%)
	Advanced and/or incurable	65 (41%)
	Do not know	25 (16%)
Time since diagnosis	0–3 months	72 (45%)
	4–6 months	29 (18%)
	7–12 months	12 (7.5%)
	More than 12 months	47 (29%)

Note: Not all rows add up to 165 due to missing data.

**Table 2:** The proportion of participants reporting receiving care for each item (*n* = 165).

	Care Received?	
	Yes *n* (%)	No *n* (%)
How you might feel physically?	155 (94%)	10 (6.1%)
Who to contact if you have any unexpected side effects between treatments?	153 (93%)	12 (7.3%)
Side-effects to watch out for?	153 (93%)	11 (6.7%)
What would happen on the first day of treatment?	150 (91%)	15 (9.1%)
The best way of managing any side effects?	150 (91%)	15 (9.1%)
What side effects mean you should get medical care urgently?	150 (91%)	14 (8.5%)
What to do to stay as well as you can during treatment?	150 (91%)	15 (9.1%)
Contacting a health professional who is located at the clinic where you are receiving treatment?	143 (88%)	20 (12%)
Contacting a health professional who is available any day of the week from 8 a.m.–8 p.m.	142 (86%)	23 (14%)
What to do after treatment?	141 (86%)	23 (14%)
How to cope with any stress or worry?	132 (80%)	33 (20%)

Note: Not all rows add up to 165 due to missing data.

**Table 3:** Results of the linear regression on actual care received (*n* = 156).

Variable	Category	Estimate of Score Difference (95% CI)	*p*-Value
Cancer type	Breast	1.20 (0.20 to 2.19)	0.1044
	Colorectal/bowel	0.98 (0.11 to 1.86)	
	Haematological/blood (e.g., lymphoma, leukaemia, myeloma)	0.65 (−0.45 to 1.75)	
	Lung	0.78 (−0.25 to 1.80)	
	Other	Reference	
Country of birth	Other	−1.37 (−2.74 to 0.00)	0.1945
	United Kingdom	−0.00 (−0.96 to 0.95)	
	Australia	Reference	
Age	Continuous	−0.02 (−0.05 to 0.00)	0.0949
Highest level of education	High school	−0.82 (−1.95 to 0.32)	0.2375
	Trade or vocational training	−1.46 (−2.89 to −0.03)	
	University degree	−1.26 (−2.59 to 0.08)	
	Primary school	Reference	
Gender	Female	−1.34 (−2.28 to −0.41) *	0.0083
	Male	Reference	
Cancer stage at diagnosis	Advanced and/or incurable	1.54 (0.37 to 2.71)	0.0594
	Early	1.28 (0.05 to 2.52)	
	Do not know	Reference	
Time since diagnosis	4–6 months	0.83 (0.10 to 1.56)	0.1513
	7–12 months	0.12 (−1.42 to 1.67)	
	More than 12 months	−0.13 (−0.98 to 0.72)	
	0–3 months	Reference	

Note: Only 156 participants had complete data for the socioeconomic/disease variables and were able to be included in the regression. * = significant at *p* < 0.05.

## Data Availability

The data presented in this study are available on request from the corresponding author. The data are not publicly available due to ethical restrictions.
